# 3D dynamic diuretic renal scintigraphy using a hybrid whole body CZT SPECT/CT camera protocol in the evaluation of acute ureteric obstruction caused by ureteric stone

**DOI:** 10.1186/s41824-024-00213-9

**Published:** 2024-09-02

**Authors:** Miguel Ochoa-Figueroa, Klara Borbely, Diana Hasselqvist, Fredrik Askling, Tobias Lööw, Firas Aljabery, Veronica Sanchez-Rodriguez

**Affiliations:** 1 Department of Clinical Physiology in Linköping, and Department of Health, Medicine and Caring Sciences, Linköping University, 581 85 Linköping, Sweden; 2 Department of Radiology in Linköping, and Department of Health, Medicine and Caring Sciences, Linköping University, Linköping, Sweden; 3 Center for Medical Image Science and Visualization (CMIV), Linköping University, Linköping, Sweden; 4 Emergency Department in Linköping, and Department of Health, Medicine and Caring Sciences, Linköping University, Linköping, Sweden; 5 Division of Urology, Department of Clinical and Experimental Medicine, Linköping University Hospital, Linköping, Sweden

**Keywords:** Stone, Ureteral obstruction, SPECT, CT, Cadmium-zinc-telluride

## Abstract

**Purpose:**

To investigate the performance of dynamic 3D diuretic renal scintigraphy using a hybrid whole body CZT SPECT/CT for the evaluation of acute ureteric obstruction in patients with urinary stone disease.

**Methods:**

20 patients who presented to the Emergency Department with acute renal colic due to urinary stone disease confirmed by means of CT were prospectively included. Three observers evaluated and graded hydronephrosis, hydroureter, perirenal stranding, and thickening of the renal fascia from the CT as well as the renal scintigraphy curves from the dynamic SPECT study. The normalized residual activity from dynamic SPECT was analysed at 16 min in all patients and at 20 min in suspected obstruction.

**Results:**

Renal scintigraphy curves showed a sensitivity of 100%, specificity of 93%, PPV 83% and a NPV 100% for obstruction, while normalized residual activity showed a sensitivity of 100%, specificity of 73%, PPV 56% and a NPV 100%. All patients presented at least 2 secondary signs of obstruction on the CT, showing a PPV of only 25% for obstruction.

**Conclusion:**

Dynamic 3D diuretic renal scintigraphy CZT SPECT/CT provides valuable functional and anatomical information from one single examination. The combination of pathological renogram curves and high normalized residual activity values provide the most valuable imaging information to determine the presence of acute ureteric obstruction. The secondary signs of obstruction observed on CT are not specific and should not be used to confirm or discard obstruction in patients with urinary stone disease.

*Trial registration*: ISRCTN15338358. Registration date 03/01/2024. Retrospectively registered. https://www.isrctn.com/ISRCTN15338358?q=miguel%20ochoa%20figueroa&filters=&sort=&offset=1&totalResults=2&page=1&pageSize=10

## Introduction

**Fig. 1:**
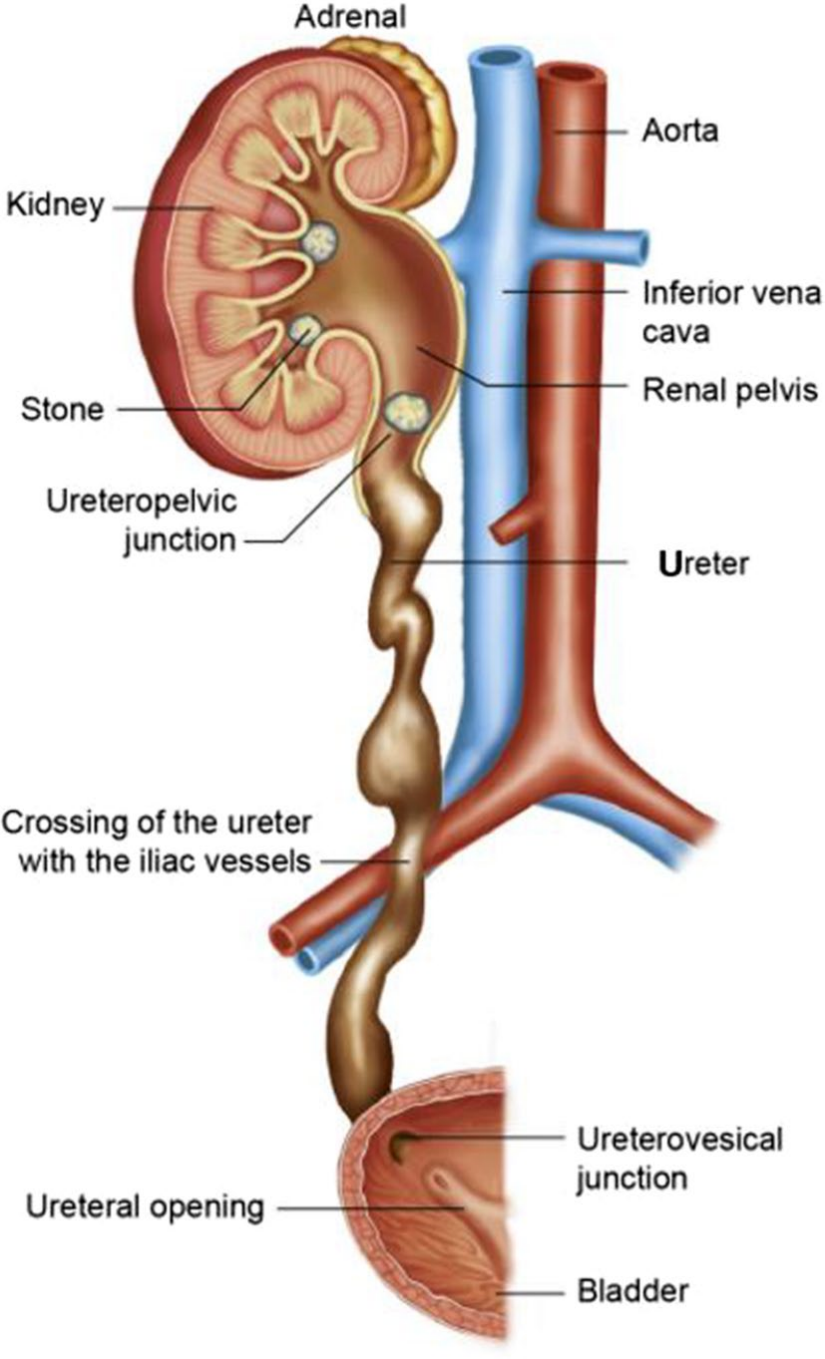
Stone former kidney showing stones in the urinary system

Abdominal pain is one of the most common reasons for Emergency Department visits, with urinary stone disease (USD) accounting for around 5% of presenting cases (Meltzer et al. 2017). USD is clinically manifested as renal colic, an acute condition usually causing nausea, vomiting, hematuria and abdominal pain, which in some cases is of such intensity that patients describe it as the most painful experience of their lifetime (Golzari et al. 2014). USD can potentially cause obstruction of the urinary system (OUS) which can lead to renal damage of varying degree. The extent of damage can be dependent on how quickly the obstruction is managed, potentially causing urinary retention, urinary tract infections, incontinence, and even chronic renal failure if untreated after a few weeks (Meltzer et al. 2017; Sfakianaki et al. 2013). Previous research has shown that patients demonstrating a definite obstruction on diuretic renal scintigraphy (DRS) must promptly undergo renal decompression to avoid renal damage (Sfakianakis et al. 2009), even if the patient is without renal colic pain which may have been supressed by analgesics. OUS can be caused by different conditions, with one of the most common causes being USD with an incidence of around 13–15% in men and 5–7% in women, and a relapse rate of 50% within 5–10 years if not managed properly (Fig. 1) (Sfakianaki et al. 2013; Sfakianakis et al. 2009; Sahlén et al. 2023; Shastri et al. 2023). It is calculated that each year $5 billion dollars are spent in the United States of America alone for related hospitalizations, stone removal procedures, and time lost from work (Sfakianaki et al. 2013). Low-dose Unenhanced Computed Tomography (UECT) has become the method of choice for evaluation of USD in an acute setting and when intervention is planned (Türk et al. 2016; Kalisz et al. 2023). However, UECT does not provide direct information regarding renal obstruction, function or urodynamics and relies on the so-called secondary signs of obstruction, such as hydronephrosis, hydroureter, perirenal stranding, thickening of the renal fascia, and unilateral renal swelling to determine if obstruction is present (Sfakianaki et al. 2013). These secondary signs of obstruction have also shown no direct relationship to OUS, with a low positive predictive value (PPV) of 56% (Sfakianaki et al. 2013; Sfakianakis et al. 2009; Sahlén et al. 2023). DRS is a widely available imaging test that can evaluate renal function and urine transit, with a previously demonstrated 90.6% PPV for obstruction and has proven to be of value in patients with acute onset of USD (Sfakianaki et al. 2013; Ochoa-Figueroa et al. 2019). The development of novel digital multi-modality equipment such as multipurpose whole body Cadmium Zinc Telluride (CZT) SPECT/CT cameras offers the possibility to perform a simultaneous CT and 3D dynamic DRS. Such cameras have been shown to significantly improve energy resolution, count rate performance, and image contrast, compared to a conventional Anger camera, providing better image quality, sensitivity, and specificity rates (Desmonts et al. 2020). This multimodality approach can lead to improved patient management by diagnosing obstruction and its cause in a single examination. Additionally, the technique also allows evaluation of normalized residual activity (NORA), which is a physiological parameter of renal drainage with the advantage of being independent for renal function, providing semiquantitative data of kidney drainage at a given moment and improving the accuracy of the test (Piepsz et al. 2000, 2002). The goal of this study was to assess the feasibility of performing dynamic 3D DRS using a novel CZT SPECT/CT camera in patients with acute renal colic and USD by utilizing a hybrid imaging approach to obtain all the information from a single examination.

## Materials and methods

### Ethical approval

Approval for the study was granted by the regional ethical board (Dnr 2019–01608). Written informed consent was obtained from all patients included in the study as demanded by the ethical board.

### Study population

Patients with two native kidneys who presented with renal colic at the Emergency Department of our Institution between February 2022 and December 2023, had a unilateral USD confirmed by UECT and accepted to participate, were included in the study.

### UECT settings

The CT scanners used were Somatom Definition Edge and Somatom Drive (both Siemens Medical Solutions, Forchheim, Germany). The scans were performed with automatic tube current modulation CARE Dose 4D, Quality Reference 38 mAs and Care kV with a reference of 120 kV. The parameters were as follows: rotation time = 0.5 s; collimation = 128 × 0.6 mm; pitch = 0.6, kernel Bf37 with Admire 3.

### CZT SPECT/CT settings and 3D dynamic DRS protocol

A VERITON CZT 360° SPECT/CT scanner (Spectrum Dynamics Medical, Caesarea, Israel) was used for all studies. Sensitivity calibration was applied to the SPECT system according to manufacturer recommendations, thus allowing absolute rather than relative quantification. DRS scans were performed according to SNMMI procedure standard/EANM practice guidelines (Taylor et al. 2018).

Imaging was performed using a dynamic protocol with a standard dose of 75 MBq (± 10%) ^99m^Tc-mercaptoacetyltriglycine i.v. (NephroMAG, ROTOP Pharmaka GmbH, Dresden, Germany). Patients were hydrated with 10 ml/kg water 1 h pre-injection of NephroMAG up to the point of imaging acquisition.

Furosemide 10 mg/ml (Orifarm Healthcare A/S, Odense, Denmark) diuretics were administrated intravenously 1 min before administration of NephroMAG, aiming to perform a F = 0 protocol (Sfakianakis et al. 2009; Taylor et al. 2018). Furosemide dose was calculated using the following formula;

$$Total\; amount \left( {{\text{ml}}} \right) = \frac{{Body \;weight \left( {{\text{kg}}} \right) \times 0.5}}{10}$$

The formula was used up to a maximum dose of 40 mg (4 ml) for 80 kg of body weight; all patients weighing > 80 kg received a standard furosemide dose of 40 mg (4 ml).

An abdominal UECT (from the diaphragm dome to symphysis pubis) was acquired using the following settings: 120 kV, 26.7 effective mAs, 20 mA with a collimation of 40 mm. A large transaxial FOV with a 500 mm scan length was used. SPECT acquisition was performed as a 360-degree 3D dynamic scan, reconstructed in a 256 matrix with 2.46 × 2.46-pixel size setting, resolution recovery, attenuation correction and 314 mm field of view (FOV). SPECT acquisition time for the SPECT was set to a minimum of 16 min (960 s) and a maximum of 30 min (1800s). Mean acquisition time was 20 min (1200 s), with the variability of acquisition durations mainly due to individual patient´s wishes to end the examination because of a need to urinate or abdominal discomfort, or as indicated by the pattern of the DRS curve.

The decision to end the procedure was made by an on-site nuclear medicine specialist.

All images were analysed using MIM software (Ver 7.3.4, Cleveland, OH, US), drawing volumes of interest (VOI) over both kidneys and the aorta on the SPECT/CT images, following the software instructions. Images were then analysed by two different readers trained in nuclear medicine and radiology, each with more than 10 years’ experience in DRS (R1 and R3) and one radiology specialist currently additionally training in nuclear medicine (R2).

### Nomenclature

All patients had the standard follow-up period and management according to current Swedish National Healthcare guidelines for kidney stone disease (Nationellt vårdprogram för stensjukdom i övre urinvägarna). Kidneys in which the managing physician decided to perform acute decompression intervention will be referred to as “obstructed kidneys” regardless of imaging findings. The contralateral healthy kidneys and kidneys which did not require acute intervention will be referred to as the “non-obstructed kidneys” since this is the status of the kidneys in all imaging scans and clinical status

### Grading secondary signs of obstruction in UECT

Image data from the UECT scan performed during the onset of renal colic in the Emergency Department was evaluated according to the grading scale used previously by Sahlén et al. (2023) with a scale of 0–3 when grading hydronephrosis, hydroureter, perirenal stranding, and thickening of the renal fascia as follows: 0 = normal, 1 = mild, 2 = moderate; and 3 = severe. Hydronephrosis was graded based on the width of the calyces, with no measurement specification, as is done in clinical practice, since there is no consensus for grading hydronephrosis on CT. The secondary signs were correlated as sums and separately to the findings in the diuretic renogram. Three observers (R1, R2, R3) independently, and later in consensus, evaluated the degree of secondary signs, blinded to the results of the diuretic renal scintigraphy and patient outcome.

### Evaluation of the DRS findings

The dynamic 3D DRS findings were analysed according to current guidelines for DRS (Taylor et al. 2018) by three observers (R1, R2, R3) independently, then later in consensus, blinded to the results of the UECT and patient outcome. The evaluation based on consensus was used in the statistical analysis.

A scale of 0–3 was used to evaluate the renal scintigraphy curves; no obstruction = 0, hydronephrosis = 1, obstruction = 2, or partial obstruction (indeterminate) = 3 (Taylor et al. 2018; Sfakianakis et al. 2000).

Quantification of the residual activity in the kidney was performed by placing a VOI around the kidney in the last frame of the dynamic 3D acquisition, independently of the duration of the study, and the activity was obtained as Bq/ml. Data in Bq/ml, rather than counts, allows for the absolute quantification of activity remaining in the kidney as opposed to a relative left vs right percentage.

### Normalized residual activity

NORA was evaluated at 16 min in all patients and additionally at 20 min in patients with obstruction. The results were graded from 1 to 5 according to previously published material (Piepsz et al. 2000, 2002).

### Statistical analysis

Inter-observer agreement was assessed through Bland–Altman plots and 95% limits of agreement for the ratio-scale indexes that were based on the imaging variables. Also, inter-observer agreement was assessed through confusion matrices and the unweighted Cohen’s kappa (κ) for the nominal-scale DRS curve variable. Inter-observer agreement was regarded as weak if Cohen’s κ was in the interval 0.40–0.59, moderate if Cohen’s κ was in the interval 0.60–0.79, strong if Cohen’s κ was in the interval 0.80–0.89, and almost perfect if Cohen’s κ was in the interval 0.90–1.00 (McHugh 2012).

Sensitivity, specificity, positive predictive values (PPV), and negative predictive values (NPV) were computed for three binary variables that were used to detect outflow obstruction: (i) a variable based on the consensus reading of the renogram curve, (ii) a variable based on NORA, (iii) a variable indicating if there were at least two secondary signs of obstruction on the CT. The outcomes on these variables were compared to the variable indicating if each patient had an outflow obstruction according to his or her final diagnosis (gold standard).

Spearman’s correlation coefficient was computed for each association between the consensus assessment of each the UECT variables, the dichotomized variable for the consensus reading of the DRS curve (normal/non-normal), and the variable for the patient’s final diagnosis (obstructed/non-obstructed). In accordance with standard practice, it was decided that α = 0.05.

Statistical analyses were performed using R statistical software version 4.1.1.

## Results

A total of 20 adult patients were included in the study, 13 were male (65%). Median age was 50 years (19–75). The mean time between UECT imaging acquisition and follow-on CZT SPECT/CT was 23 h.

Demographics, imaging, and clinical findings for patients with obstructed and non-obstructed kidneys can be seen in Tables 1 and 2 respectively.

**Table 1:** Patients without OUS according to final diagnosis and management

Patient number	Sex	Age	Secondary signs at UECT scan	Side of stone location L/R	Maximum size of stone (mm)	HU stone	Location of stone in urinary system UECT (*)	DRS curve pattern of affected kidney	DRS split function (%) L/R	DRS *T*_*max*_ (seconds) L/R	DRS NORA values L/R	VOI kidney quantification in Bq/ml L/R	Serum creatinine	Follow up
1	M	69	2 = HN, HUR	R	4	550	U1	0	47/53	138/171	0.4/0.6	383/485	85	EELL
2	M	25	2 = HN, HUR	R	5	850	U1	0	52/48	238/272	0.5/0.5	2894/3315	68	EELL
3	M	29	2 = HN, HUR	R	2	250	U3	1	58/42	122/1412	0.5/3.1	1434/9602	83	Spontaneous passage
4	F	22	2 = HN, HUR	R	2	230	U3	1	60/40	181/1783	0.4/3.1	686/8940	79	Spontaneous passage
5	F	49	2 = HN, HUR	L	5	1000	U3	0	50/50	195/111	0.7/0.3	2367/1484	65	Spontaneous passage
7	M	65	3 = HN, HUR, FS	R	5	950	U3	1	57/43	284/660	0.7/2.2	9687/29,453	80	Spontaneous passage
8	F	29	2 = HN, HUR	L	4	450	3	3	51/49	400/150	1.8/0.3	28,132/12,326	70	Spontaneous passage
10	M	32	2 = HN, HUR	L	3	200	U1	0	50/50	219/318	0.5/0.5	9629/12,757	79	Spontaneous passage
11	M	62	4 = HN, HUR, FS, TRS	L	9	1000	U3	0	41/59	120/240	0.3/0.4	6754/9572	79	ESWL
12	M	49	4 = HN, HUR, FS, TRS	L	4	250	U3	0	48/52	310/300	0.9/1	8957/14,781	104	Spontaneous passage
13	F	64	2 = HN, HUR	R	5	700	U3	0	51/49	100/100	0.4/0.4	14,744/13,130	61	Spontaneous passage
14	F	19	2 = HN, HUR	R	5	650	U1	0	50/50	164/285	0.6/0.8	32,656/44,390	72	EELL
15	M	75	4 = HN, HUR, FS, TRS	L	7	1100	U1	0	44/56	280/320	0.9/1	13,330/14,572	66	EELL
16	M	80	4 = HN, HUR, FS, TRS	R	7	550	U2	0	47/53	200/200	0.7/0.9	12,329/18,858	138	EELL
19	F	50	2 = HN, HUR	R	8	660	U3	0	57/43	84/106	0.9/0.7	7436/15,266	80	ESWL
Mean (SD)		47.9 (19.9)	2.6 (0.87)		5 (2)	626 (292)			47 (4.3) *Value for affected kidney	434 (480) *Value for affected kidney	0.7/1 (0.3/0.9)	151,418/208,931	80.6 (18.3)	

L = left. R = right. HU = Hounsfield Units. HN, hydronephrosis; HUR, hydroureter; FS, fat stranding; TRS, thickening of the renal fascia. * = Localization of the stone in the urinary system according to Sugino et al. (2020). U0 = excreted stone. DRS curves no obstruction = 0, hydronephrosis = 1, obstruction = 2, indeterminate = 3. Serum creatine levels measured at the emergency department (Females 45–90 μmol/L. Males 60–105 μmol/L). EELL = Elective endoscopic laser lithotripsy. ESWL = Extracorporeal shock wave lithotripsy

**Table 2:** Patients with OUS according to final diagnosis and management

Patient number	Sex	Age	Secondary signs at UECT scan	Side of stone location L/R	Maximum size of stone (mm)	HU stone	Location of stone in urinary system UECT (*)	DRS curve pattern of affected kidney	DRS split function (%) L/R	DRS *T*_*max*_ (seconds) L/R	DRS NORA values L/R 16 min	DRS NORA values L/R 20 min	VOI kidney quantification in Bq/ml L/R	Serum creatinine
6	F	60	2 = HN, FS	R	3	500	U2	2	66/34	192/1593	0.4/2.7	0.3/2.8	13,547/32,671	104
9	M	60	2 = HN, HUR	L	7	1300	U1	3	36/64	721/175	2/0.5	1.9/0.4	34,270/10,663	123
17	M	57	4 = HN, HUR, FS, TRS	L	7	950	U1	2	44/56	316/98	1.4/0.3	1.4/0.3	29,967/11,590	162
18	M	36	2 = HN, HUR	R	4	600	U1	2	62/38	190/1223	0.5/3.1	0.3/3.4	13,952/66,660	129
20	M	59	3 = HN, HUR, FS	R	6	770	U1	3	51/49	116/333	0.5/1.7	0.5/1.6	10,781/30,359	123
Mean (SD)		54.4 (9.26)	2.6 (0.8)		5.4 (1.62)	824 (283)			40 (5.52) *Value for affected kidney	837 (501) *Value for affected kidney	2.2 (0.6) *Value for affected kidney	2.2 (0.7) *Value for affected kidney	102,517/151,943	128 (18.8)

L = left. R = right. HU = Hounsfield Units. HN, hydronephrosis; HUR, hydroureter; FS, fat stranding; TRS, thickening of the renal fascia. * = Localization of the stone in the urinary system according to Sugino et al. (2020). U0 = excreted stone. DRS curves no obstruction = 0, hydronephrosis = 1, obstruction = 2, indeterminate = 3. Serum creatine levels measured at the emergency department (Females 45–90 μmol/L. Males 60–105 μmol/L). EELL = Elective endoscopic laser lithotripsy. ESWL = Extracorporeal shock wave lithotripsy

### UECT

The median sum of points for secondary signs observed on CT was 4.00 (range = 2.00–6.00).

All patients presented at least 2 secondary signs of obstruction on the CT, showing a PPV of only 25% for obstruction. There was a strong and significant correlation between the assessments of hydronephrosis and the patients’ final diagnoses (*r*_*s*_ = 0.47, *p* = 0.037), but there were no significant correlations between any of the other secondary signs of obstruction and the patients’ final diagnoses (all *r*_*s*_ < 0.35 and all *p* > 0.15).

**Fig. 2:**
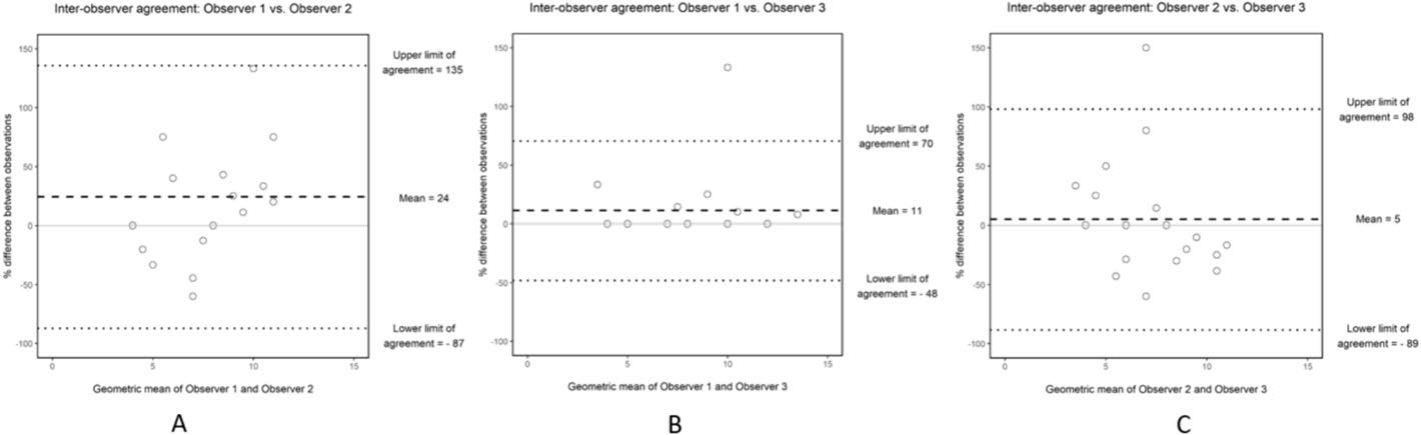
**A** Bland–Altman plot for the total score of the CT assessments made by Observer 1 and Observer 2. The mean relative difference between the assessments made by Observer 1 and Observer 2 was 41 percent, suggesting that Observer 1 on average reported more severe CT assessments than Observer 2 by a factor of 1.41. The limit of agreement ranged from − 90 to 171, which indicated that the differences between the assessments made by the observers varied greatly. **B** Bland–Altman plot for the total score of the CT assessments made by Observer 1 and Observer 3. The mean relative difference between the assessments made by Observer 1 and Observer 3 was 13 percent, suggesting that Observer 1 on average reported more severe CT assessments than Observer 3 by a factor of 1.13. The limit of agreement ranged from − 48 to 74, which indicated that the differences between the assessments made by the observers varied considerably. **C** Bland–Altman plot for the total score of the CT assessments made by Observer 2 and Observer 3. The mean relative difference between the assessments made by Observer 2 and Observer 3 was -6 percent, suggesting that Observer 2 on average reported less severe CT assessments than Observer 3 by a factor of 0.94. The limit of agreement ranged from − 94 to 83, which indicated that the differences between the assessments made by the observers varied considerably

The limits of agreement for grading of the secondary signs of obstruction observed on CT are presented in Fig. 2a–c. There was discrepancy between the observers´ results. For almost all measurements, one observer had a result with higher values compared to the other two observers.

### 3D dynamic DRS

There were no significant correlations between normal or abnormal findings on the DRS curve and the secondary signs of obstruction assessed through UECT (all *r*_*s*_ < 0.35 and all *p* > 0.10).

Renal scintigraphy curves showed a sensitivity of 100%, specificity of 93%, PPV 83% and a NPV 100% for obstruction based on consensus reading.

Assessments of the DRS curves by the different readers are presented in Tables 3, 4 and 5.

**Table 3:** Assessments of renogram curves: Confusion matrix for Observer 1 and Observer 2

		Observer 2			
		Normal	Hydronephrosis	Outflow obstruction	Partial obstruction
Observer 1	Normal	11	0	0	0
	Hydronephrosis	0	1	1	1
	Outflow obstruction	0	1	3	1
	Partial obstruction	0	0	0	1

Cohen's kappa = 0.68 (95% CI, from 0.44 to 0.92), which suggests moderate agreement between the observers. The observers fully agreed upon which renogram curves were normal and non-normal, respectively. However, in several instances, the observers disagreed upon the categories to be applied to the renogram curves when they were non-normal

**Table 4:** Assessments of renogram curves: Confusion matrix for Observer 1 and Observer 3

		Observer 3			
		Normal	Hydronephrosis	Outflow obstruction	Partial obstruction
Observer 1	Normal	11	0	0	0
	Hydronephrosis	0	3	0	0
	Outflow obstruction	0	0	3	2
	Partial obstruction	0	0	0	1

Cohen's kappa = 0.84 (95% CI, from 0.65 to 1.00), which suggests strong agreement between the observers. The observers fully agreed upon which renogram curves were normal and non-normal, respectively. However, in two instances, the observers disagreed upon the categories to be applied to the renogram curves when they were non-normal

**Table 5:** Assessments of renogram curves: Confusion matrix for Observer 2 and Observer 3

		Observer 3			
		Normal	Hydronephrosis	Outflow obstruction	Partial obstruction
Observer 2	Normal	11	0	0	0
	Hydronephrosis	0	1	0	1
	Outflow obstruction	0	1	3	0
	Partial obstruction	0	1	0	2

Cohen's kappa = 0.76 (95% CI, from 0.54 to 0.98), which suggests moderate agreement between the observers. The observers fully agreed upon which renogram curves were normal and non-normal, respectively. However, in several instances, the observers disagreed upon the categories to be applied to the renogram curves when they were non-normal

NORA values for all obstructed kidneys ranged from 1.4 to 3.1 at 16 min and from 1.4 to 3.4 at 20 min with a mean value of 2.2 at both time points. For non-obstructed kidneys NORA values ranged from 0.3 to 3.1 at 16 min with a mean value ranging from 0.7 to 1.0. NORA showed a sensitivity of 100%, specificity of 73%, PPV 56% and a NPV 100% for obstruction.

Quantification of the residual activity in the kidney during the last SPECT frame is presented in Table 1 and 2.

## Discussion

**Fig. 3:**
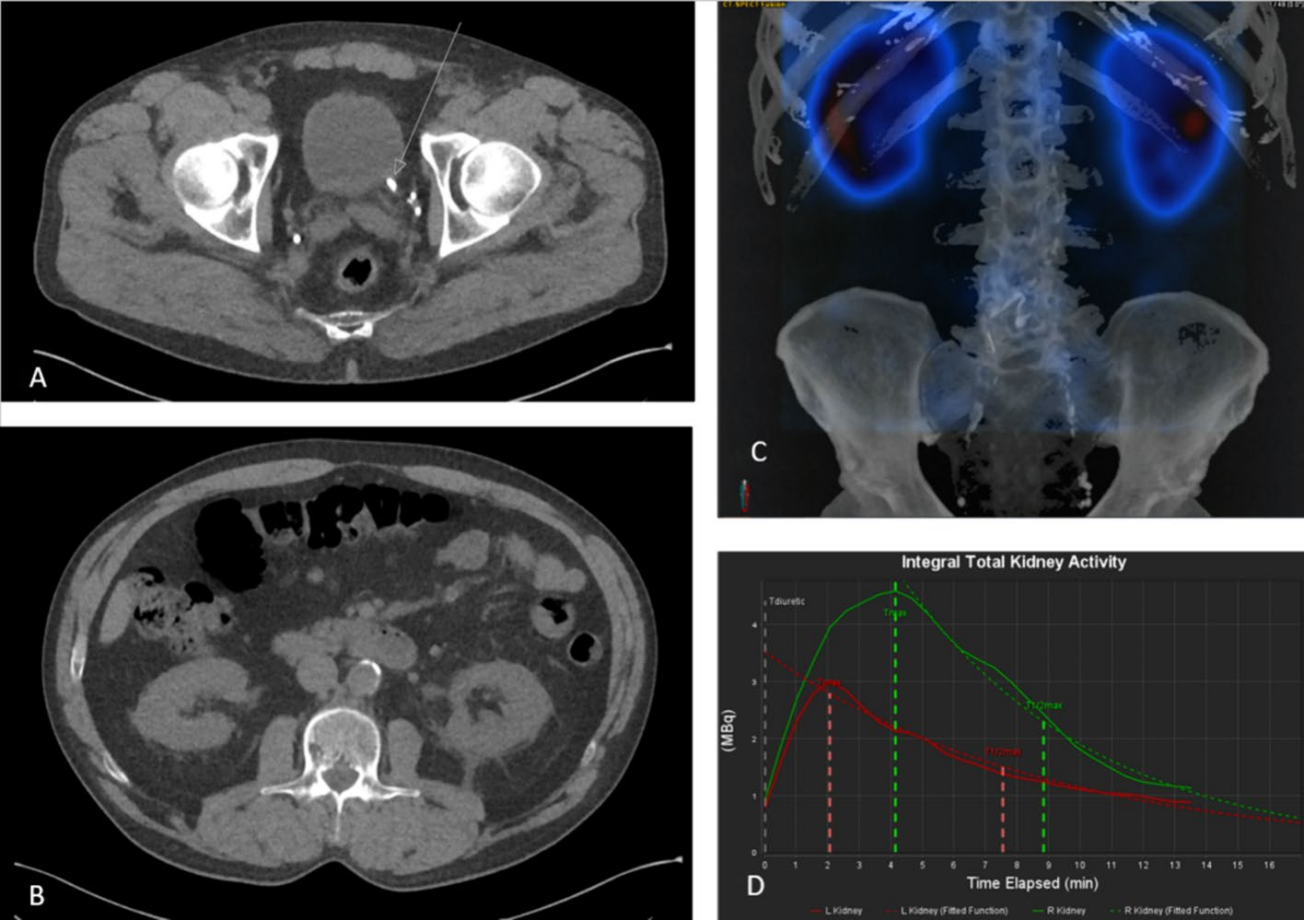
Images from case 11 in Table 1. The patient presented to the Emergency Department with renal colic symptoms, left flank pain and haematuria. **A** Axial UECT shows a stone in the left ureter close to the ostium with a diameter of 9 × 5 mm (arrow). **B** Axial UECT at kidney level shows hydroureter, fat stranding and thickening of the renal fascia in the left kidney. **C** 3D SPECT/CT reconstruction showing both kidneys with similar activity. **D** Bilateral falling renogram curves, red curve left kidney and green curve right kidney

**Fig. 4:**
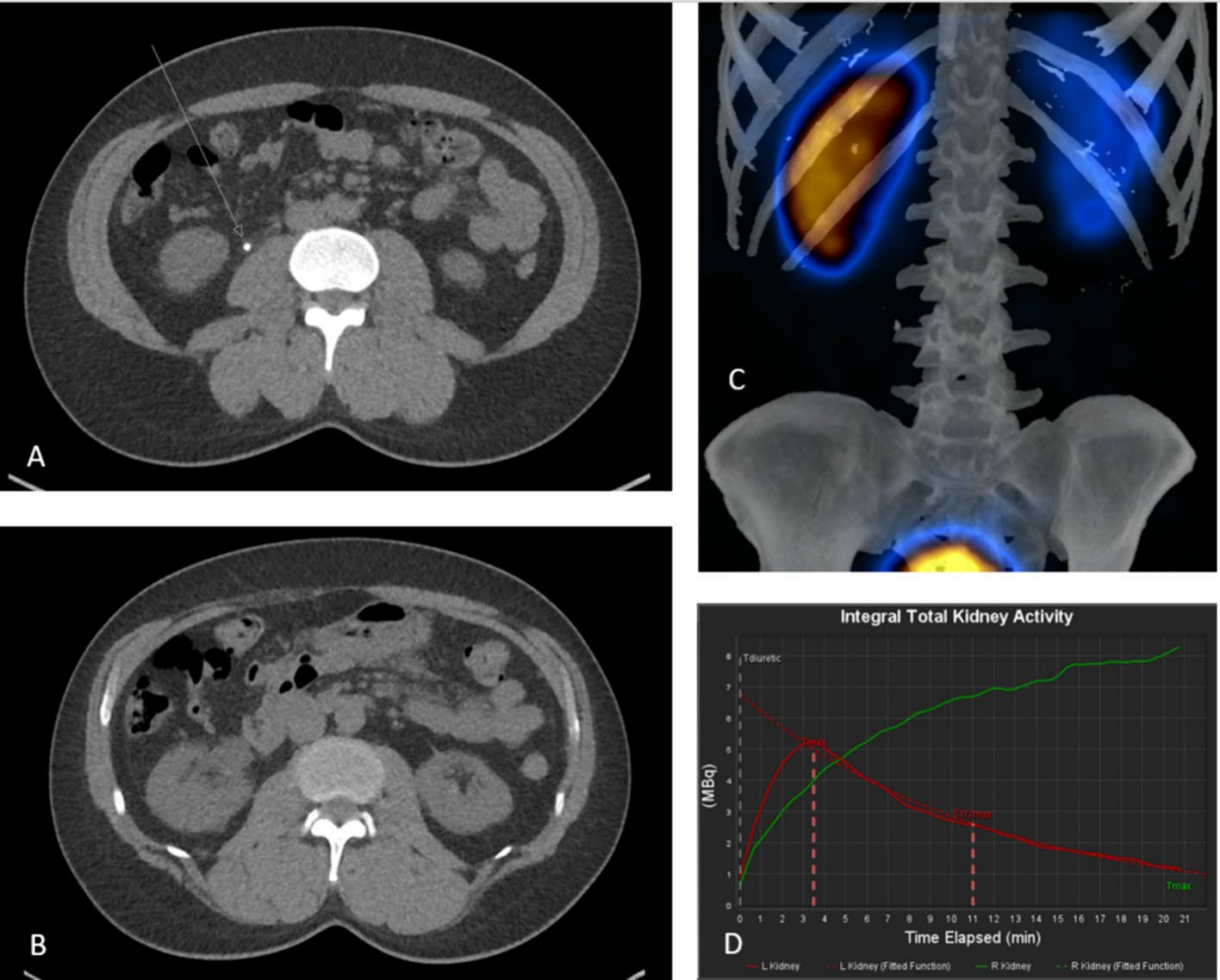
Images from case 18 in Table 2. The patient presented to the Emergency Department with renal colic symptoms, right flank pain and haematuria. **A** Axial UECT shows a stone in the proximal right ureter a maximum diameter of 3 mm (arrow). **B** Axial UECT at kidney level shows hydronephrosis of the right kidney. **C** 3D SPECT/CT reconstruction showing high tracer concentration in the right kidney while the left kidney has an adequate tracer elimination. **D** Renogram curves showing a falling curve from the left kidney (red curve) and an obstructive curve from the right kidney (red curve)

We performed a dynamic 3D renogram in patients with renal colic secondary to USD using a novel whole body CZT SPECT/CT camera, and to the authors knowledge, to date there is no published data within the scientific literature which utilizes this type of equipment and protocol in this patient population. Compared to a conventional renogram, this novel technical advantage allows visual assessment of the complete renogram examination over time in 3D imaging, as well as performing volume quantifications of the kidney activity at any time point of the SPECT acquisition. In the current work we have performed the volume quantifications in the last frame of the SPECT imaging since this time point visually shows the biggest differences between obstructed and non-obstructed kidneys and the quantification confirms values at least double in kidneys with obstruction and hydronephrosis compared to those with no obstruction (Tables 1 and 2). Additionally, this hybrid approach provides entire information from the CT component and the DRS in a single examination, demonstrating that this imaging tool could be of value for clinical practice within this patient population. CT has been used in patients with suspected USD since the mid 1990’s when Smith et al. (1995) showed that this imaging technique could be of benefit for these patients since it demonstrated sensitivity and specificity values ranging around 95% for kidney stone detection (Brisbane et al. 2016). Additionally, CT offers other advantages in the acute context, such as providing a fast diagnostic examination which can be performed in just a few minutes to reach a direct diagnosis by visualizing a stone and excluding other causes of abdominal pain. CT also provides the location, size, and an approximation of the composition of a stone; all valuable information which can determine the management of the patient (Türk et al. 2016; Kalisz et al. 2023). It is generally considered that a stone < 5 mm in diameter will not cause obstruction and will spontaneously pass through the urinary tract, but those > 6 mm can potentially cause obstruction and may require some type of intervention (Türk et al. 2016; Kalisz et al. 2023; Preminger et al. 2007; Phipps et al. 2010). The present study includes patients presenting with stones varying in size from 2 to 9 mm, however the size of the stone was not found to be a determinant for obstruction (Tables 1, 2 and Figs. 3, 4). The exclusion of obstruction is important in this patient population, not least as recovery of renal function is highly reliant on prompt treatment. And if untreated, renal obstruction can lead to irreversible permanent renal damage, and potentially to chronic renal failure, as well as other potential comorbidities such as sepsis, stricture, chronic tubulointerstitial disease, urinary retention, chronic/recurrent urinary tract infections, incontinence, and complications from long term catheter use (Preminger et al. 2007; Beckie 2009). Further, reported studies have shown that the initial impressions of obstruction by emergency department physicians (using clinical, laboratory and UECT results) were correct less than half of the time (Hsiao et al. 2007), thus suggesting a potentially high rate of either delayed treatment or non-treatment of renal obstruction.

Pioneers in CT imaging observed other imaging findings when interpreting the images of patients with USD (Smith et al. 1996; Dalrymple et al. 2000) such as hydronephrosis, hydroureter, fat stranding and thickening of the renal fascia, calling these findings as secondary signs of obstruction. Later research which performed UECT followed by conventional DRS found no correlation between the secondary signs of obstruction and OUS, showing a clear superiority of DRS compared to UECT for the diagnosis of OUS (Sfakianaki et al. 2013). Recent research (Sahlén et al. 2023) explores the correlation between secondary signs of obstruction and changes in the renal parenchymal volume of kidneys during obstruction due to KSD, finding no correlation between them. In our study we found that all 20 patients presented at least 2 secondary signs of obstruction on the CT images, however only 5 patients had renal obstruction secondary to USD. Previous studies have shown that the secondary signs of obstruction on CT have a low PPV of 56% for obstruction (Sfakianaki et al. 2013; Sfakianakis et al. 2009), and in our study we found an even lower PPV value of only 25%. On the other hand, the functional and hydrodynamic changes observed in the DRS, such as the DRS curve, performed better for the diagnosis of OUS, as can be seen in our results section and this is in line with previously published data (Sfakianaki et al. 2013; Sfakianakis et al. 2009, 2000; Taylor et al. 2018). Studies which have performed DRS and UECT in the acute setting in this patient population have shown that DRS can demonstrate obstruction in cases with negative UECT scans due to translucent stones (Sfakianakis et al. 2009).

Another valuable parameter from the DRS is NORA. Hitherto, Piepsz et al. (2002) have evaluated NORA in patients with a normal renogram and in patients with a dilated but unobstructed kidney, using furosemide 20 min after commencing the renogram, starting a new acquisition of 15 min. NORA values were evaluated at 20 min and at the end of the furosemide test (around 15 min). In adults with normal renal function 40 mg of furosemide produces maximal diuresis with urine flow rates reaching approximately 20 mL/min within 3–6 min (Piepsz et al. 2002 Jan), and a peak diuretic effect between 10 and 30 min after I.V. administration (Oh and Han 2015). In the present study we used furosemide aiming to perform a F = 0 protocol and performed the NORA evaluation at 16 min (Taylor et al. 2018), finding that all kidneys without stones had a NORA value of ≤ 1 and mean value of 0.54 (SD 0.21). As can be seen in Table 2, all obstructed kidneys presented NORA values of ≥ 1.4 and above. Four patients with no obstruction presented elevated NORA values ranging from 1.8 to 3.1, as well as increased time to reach maximum counts (*T*_*max*_). However, none of these patients presented a clear obstructive curve pattern in the renogram nor elevated levels of serum creatine. Additionally, the size of kidney stones ranged from 2 to 5 mm, and all had spontaneous passage (Table 1).

A limitation of the present work is the relatively small number of patients included during the ongoing study period. Patient recruitment was difficult as most patients were already in pain and suffering from an acute renal colic episode, therefore were very reluctant to potentially experience further abdominal pain or discomfort associated with the administration of intravenous furosemide for performing the dynamic 3D DRS SPECT/CT study. Additionally, the COVID-19 pandemic caused by the severe acute respiratory syndrome coronavirus (SARS-CoV-2) was still ongoing when the team began to recruit patients, thus contact with patients was severely restricted and a notable limitation on recruitment of patients into the study.

Despite all the information on renal function and anatomy provided by 3D dynamic SPECT/CT imaging (Tables 1 and 2), there seems to be no single measurement that can serve to differentiate between non-obstructed and obstructed kidneys. From our findings using 3D DRS CZT SPECT/CT, it seems reasonable to state that once a stone and hydronephrosis are visualized in the urinary tract by means of CT, the combination of pathological findings in; the renogram curves and NORA values from the DRS, together with serum creatinine, can provide the most accurate information on OUS in patients with USD. The secondary signs of obstruction observed on CT are not specific of OUS and should not be used to confirm or discard obstruction in clinical practice in patients with USD. The novel quantification of the SPECT imaging, placing a VOI over the kidney in the last frame image shows clear differences in the residual activity in kidneys with obstruction and hydronephrosis compared to non-obstructed or non-hydronephrotic kidneys and may help to differentiate these findings in the renogram. Additional research of absolute (Bq/ml or SUV) kidney quantification is needed in order to confirm our findings and further assess kidney function using this novel quantification method.

## Conclusion

Dynamic 3D DRS using a whole body CZT SPECT/CT camera provides valuable functional and anatomical information from one single examination, which is useful for the diagnosis and management planning in patients with USD. Further studies are warranted to evaluate the potential value of this hybrid imaging methodology in USD.

## Data Availability

The datasets generated during the current study are available from the corresponding author on reasonable request.
